# Intrathecal Access Through Suboccipital Port in Patients With Spinal Muscular Atrophy and Complex Spines: Case Series and Technical Note

**DOI:** 10.7759/cureus.9525

**Published:** 2020-08-02

**Authors:** Diem Kieu Tran, Vaibhavi Shah, Michael G Muhonen

**Affiliations:** 1 Neurosurgery, University of California Irvine, Orange, USA; 2 Neurosurgery, Childrens Hospital Orange County, Orange, USA; 3 Pediatric Neurosurgery, Children's Hospital of Orange County, Orange, USA; 4 Neurosurgery, Children's Hospital of Orange County, Orange, USA

**Keywords:** sma, csf, spinraza, nusinersen, neuromuscular, intrathecal

## Abstract

Introduction

Many patients with the spinal muscular atrophy (SMA) have complex spinal anatomy, secondary to thoraco-lumbar spinal fusions. Their fragile musculoskeletal anatomy potentiates limb and joint injury if conventional spinal fluid access modalities are utilized. This creates a challenge when attempting to deliver intrathecal medications such as nusinersen (Spinraza®). Catheter placement in the cervical subarachnoid space with a caudally directed tip is potentially beneficial. This article describes our experience with Spinraza injections into the thecal space through a suboccipital port. This allowed for simple, chronic, and reliable cerebrospinal fluid (CSF) aspiration and intrathecal injections.

Methods

A total of 15 patients with SMA and complex spinal anatomy were implanted with a cervical subarachnoid catheter, connected to a suboccipital access port. We retrospectively reviewed the charts of these patients for clinical outcomes and complications. All patients then underwent serial port cannulation, aspiration of CSF, and injection of Spinraza following standard manufacturer dosage guidelines.

Results

The age range was 3 to 49. Two had type-1 SMA, 10 had type-2 SMA, and three had type-3 SMA. We were able to successfully cannulate the port, aspirate CSF, and inject Spinraza during all access attempts. Two incidents of subcutaneous CSF leaks were resolved through reoperation and one incident of transient CSF leak was resolved without surgical repair.

Conclusion

Patients with SMA requiring intrathecal injections of Spinraza can be treated safely and efficiently with this novel implantation technique. The complication rates are low and the injection time is dramatically lower than with conventional injection techniques.

## Introduction

Spinal muscular atrophy (SMA) is a rare autosomal-recessive progressive neuromuscular disorder distinguished by the degeneration of spinal motor neurons and weakness in corresponding myotomes. Nusinersen, marketed as Spinraza®, is approved for the treatment of SMA. Lifelong, periodic intrathecal bolus injections of Spinraza® are required to stabilize this disease [1]. This presents a clinical challenge, as many patients with SMA present with complex spinal pathology, including extensive spinal fusions. These anatomical challenges can complicate access to the intrathecal space. 

This vast and varied array of cerebral spinal fluid (CSF) access techniques can lead to inconsistent drug delivery and potentially variable clinical results. This led us to pursue a technique that would allow for simple, safe, and reliable access of the subarachnoid space that would, in turn, allow for painless, rapid, and safe Spinraza® injections. Because this patient population has a very fragile body habitus, and are often ventilated, it was a major goal of the implantation to develop an injection procedure that would not require the patient to be moved from the sitting position during the procedure.

At our institution, we have begun implanting the Angiodynamics Vortex® flow Smart Port® (Angiodynamics, Latham, NY), attached to a spinal catheter placed into the cervical spine for Spinraza® injections. The implant construct consists of a Medtronic intrathecal catheter (Medtronic, Minneapolis, MN) connected to the port. This port is normally used in cancer patients for vascular access [2]. However, this off-label use allows for easy access to the subarachnoid space. This vortex design ensures that all the injected medication passes through the port, which provides efficient delivery into the subarachnoid space. Implanting this device into the cervical spine allows for the gravitational movement of the Spinraza® treatment within the intrathecal space. This article describes our initial experience with this new method.

## Materials and methods

Patient selection

A total of 15 patients were included in this retrospective review study between August 2017 and April 2020 (see Table 1). To be included in the study, the patient must have a diagnosis of SMA and undergone port placement for Spinraza® injections. Subjects must also have at least six months of follow up. The patients were aged 3-49 years old (13 females, two males). Two patients had type-1 SMA, 10 individuals had type-2 SMA, and three individuals had type-3 SMA. SMN2 copy numbers were as follows: six patients with two copies, eight patients with three copies, and one patient with four copies. This study was approved as a retrospective clinical study by the Institutional Review Board (IRB) at the authors’ institution. Consent was not needed according to our IRB protocol for retrospective reviews.

**Table 1:** Overview of patient demographics and SMA history SMA - spinal muscular atrophy

Patient	Sex	Age	SMA type	SMN2 copies
1	F	29	2	2
2	F	14	2	3
3	F	40	2	3
4	M	49	3	4
5	F	45	2	3
6	F	16	2	2
7	F	19	3	3
8	F	20	3	3
9	F	27	2	3
10	F	9	1	2
11	F	26	2	2
12	F	7	2	2
13	F	6	1	3
14	M	7	2	3
15	F	3	2	2

Surgical technique

**Figure 1:**
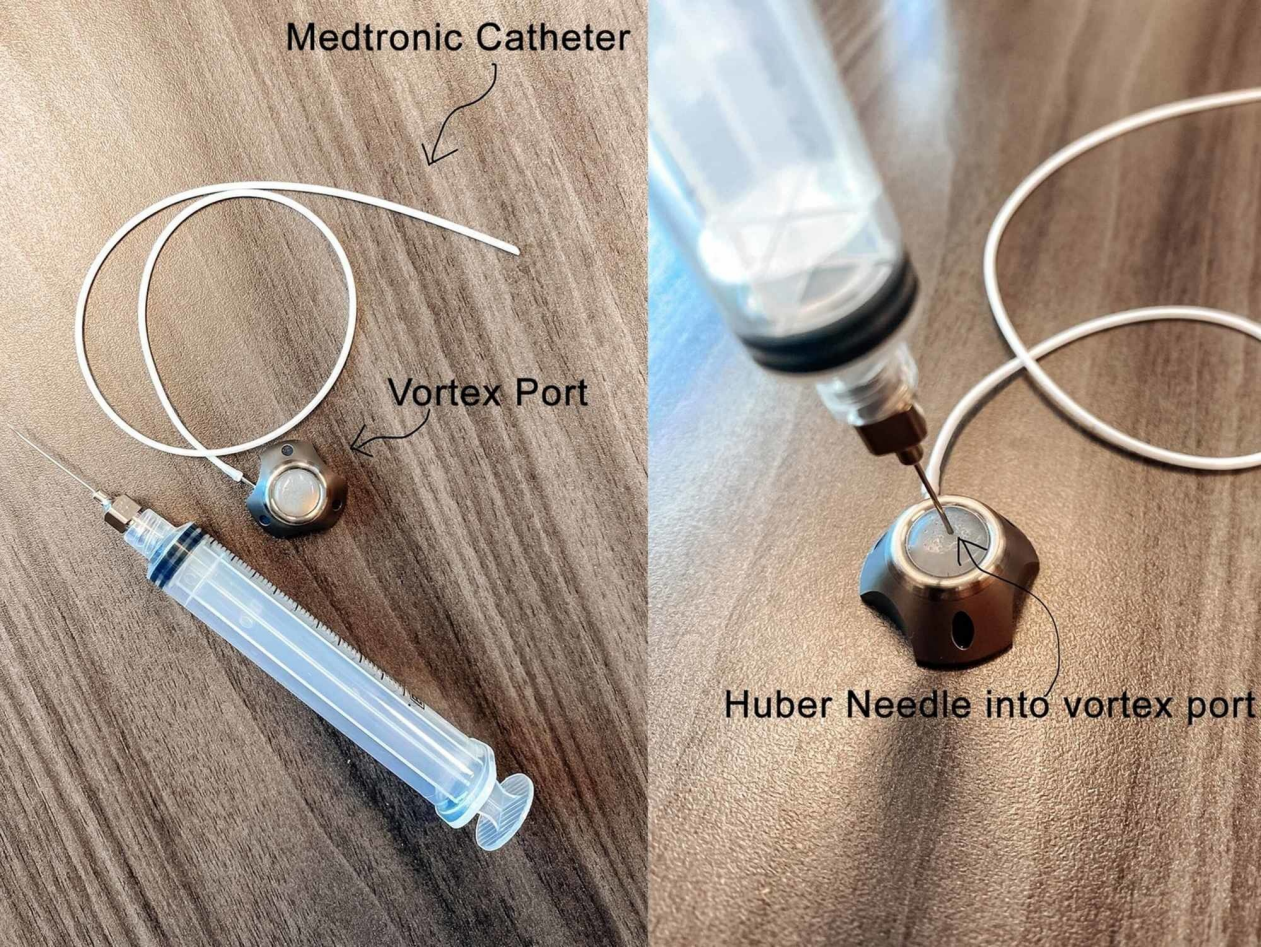
Sample of Vortex port along with Medtronic catheter and Huber needle

**Figure 2:**
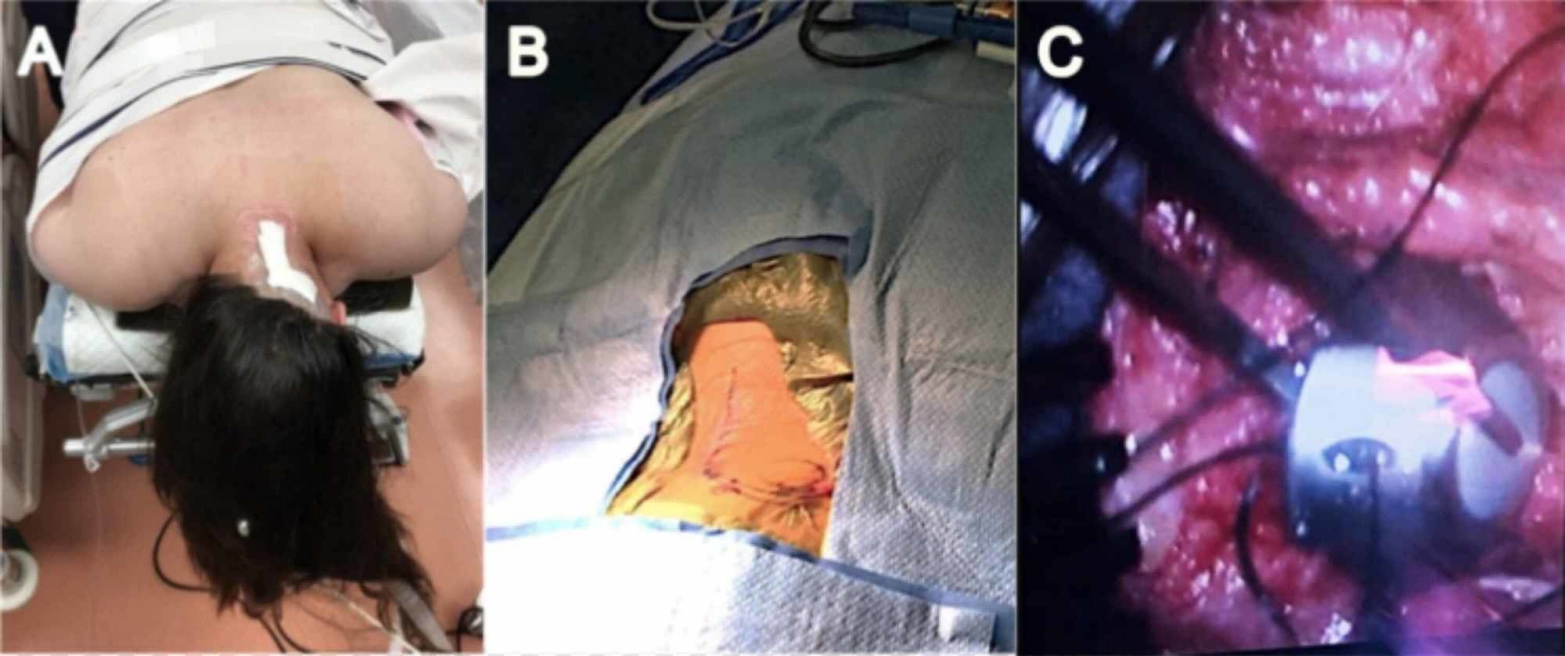
Insertion and anchoring procedure A: patient positioned prone on the operating table. B: the incision is marked. C: insertion and anchoring of the Vortex port.

The patient is positioned prone with the head on a foam form or Mayfield pins depending on their age. A standard cervical laminotomy incision is created and subperiosteal dissection is then done to identify the lamina. The hemi-laminotomy is performed at the C3 or C4 level. Once the dura is exposed, a tiny durotomy the size of the catheter is created. A Medtronic subarachnoid catheter is passed approximately at least 3 cm caudally into the subarachnoid space. Passing the catheter at least 3 cm to minimize the risk of catheter withdrawal out of the subarachnoid space. Once CSF flow was confirmed, the distal end of the catheter is tunneled laterally toward the suboccipital region. A rubber boot is then used to anchor the catheter over the epidural space. The distal end of the catheter is then connected to an Angiodynamics port, and a watertight seal is created at the attachment site using a metal snap-on connector provided with the port. Confirmation of CSF flow is done by cannulating the port with a 22-gauge Huber needle (Figure 1). The port is then anchored onto the fascia in the suboccipital region using 2-0 silk suture at three separate points. The wound is closed in layers with a fascial layer being placed over the port (Figure 2A-C). 

Injection technique

**Figure 3:**
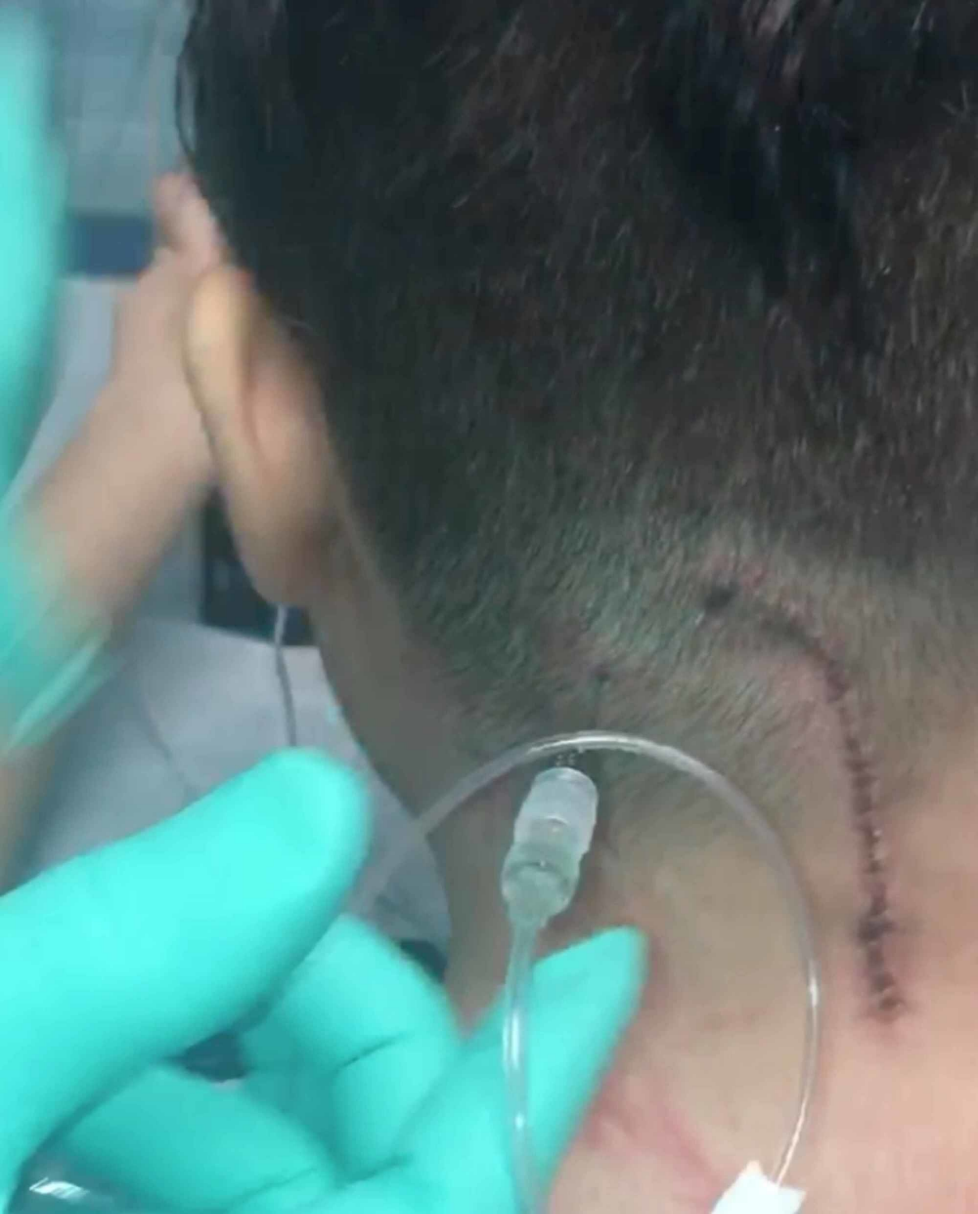
Process of medication injection A 22-gauge Huber needle attached to a syringe is used to cannulate the port.

With the patient sitting upright, the port is identified under the skin. The area is prepped and draped in the usual sterile fashion. A 22-gauge Huber needle is used to cannulate the port. Six mL of the patient’s CSF is aspirated from the port system to verify that the catheter tip is within the subarachnoid space. The medication is then injected through the port with the standard dosing, and then one mL of the patient’s CSF is used to flush through the port (Figure 3). The needle is then removed, and a small bandage is placed over the injection site. All injections were done with the patients sitting upright, usually in their wheelchairs. All 122 CSF aspiration and drug injection procedures were completed in an average time of 10 minutes, and no anesthetic was utilized.

Data collection

The clinical data of subjects who underwent this procedure from August 2017 to April 2020 were retrospectively collected and analyzed. Data for the following variables were collected: age, sex, type of SMA, operative times, number of injections, clinical outcome, and complications.

## Results

Device function, procedure safety, and ease of aspiration of CSF and injection of Spinraza® were evaluated. The results can be found in Table 2. The port was utilized in all patients within two weeks of the procedure. A total of 122 injections were administered in the 15 patients through the suboccipital port in a clinical setting, with an average of eight injections per patient. The first injection began one to two weeks post-operatively, with the first three injections 14 days apart each, the fourth injection 30 days after the third injection, and subsequent injections every four months. Some patients had already started the Spinraza® injections prior to port implantation, therefore did not require frequent start up injections during the first three months. In all 122 procedures, there was a successful aspiration of CSF and easy injection of Spinraza® into the subarachnoid space. None of the ports or catheters have failed. No infections or abnormal port movements were noted postoperatively. All patients report stabilization of motor function with some reporting significant gains during the first 24 months of the study. The functional improvements will be reviewed at a later date when more long-term data are available. The complication rates were low with three patients experiencing CSF leaks, and only two required surgical revision. Average anesthetic and preparation time, including patient positioning, was 124 minutes. The average surgical time was 58 minutes. All 15 patients were stable in the postoperative setting, with no severe complications post-operatively.

**Table 2:** Overview of patient injections and outcome * Patient experienced transient CSF leaks that was self-limiting and resolved. ** Patients who experienced postoperative CSF requiring take back for repairs. CSF - cerebral spinal fluid

Patient	Time after port implantation (days)	Number of injections	Total anesthesia time (minutes)	Total surgical time (minutes)
1	1014	12	112	49
2*	1000	12	125	61
3**	974	12	131	64
4	862	11	118	58
5	762	10	141	51
6	743	10	102	75
7	568	8	117	52
8	398	7	135	70
9	351	6	161	75
10	349	6	105	46
11**	279	6	132	68
12	258	6	142	63
13	215	5	115	55
14	321	6	119	58
15	362	6	98	46

## Discussion

SMA is a progressive motor neuron disease that typically manifests in children with a prevalence of ∼ 1 in 10,000 live births [3]. Mutations or deletions in survival motor neuron (SMN1) cause a reduction in survival motor neuron protein (SMN) expression, which results in the degeneration of lower motor neurons [4]. Physiologically, both copies of the SMN1 gene are disturbed in an affected individual [5]. To compensate for this loss of SMN protein, SMN2, a homologous gene, produces the SMN protein. The extent to which the SMN protein is created typically correlates with clinical outcome and a less severe phenotype is proportional to the number of copies of SMN2 [6].

Spinraza® was FDA approved in December 2016 as the first and only treatment for patient with SMA [7]. It is an antisense oligonucleotide drug that modifies pre-messenger RNA splicing of the SMN2 gene and augments exon 7 inclusion in SMN2, in turn producing a more stable protein that can withstand some degradation [2]. Many studies have been published that show the safety and efficacy of Spinraza® in SMA patients [2, 8-11]. However, to maintain the SMN protein production, patients need lifelong periodic injections. Many techniques to access the subarachnoid space in this patient population have been utilized. This includes standard lumbar punctures through a "window" created in the spinal fusion, transforaminal cannulation, trans-sacral hiatus approaches, implantation of spinal catheters, cervical and suboccipital punctures, and even ventricular cannulations [12].

This novel construct simplifies intrathecal injection procedures in patients with complex spinal anatomy and is potentially a more simple, effective administrative modality for other intrathecal treatments. The surgery itself is relatively simple and does not require long surgical times. Given that the majority of SMA patients are wheelchair-bound, this suboccipital port access allows the administration of the drug to be faster and easier with a rostral injection site, obviating the need for excessive adjustment of the patient's sitting position.

**Figure 4:**
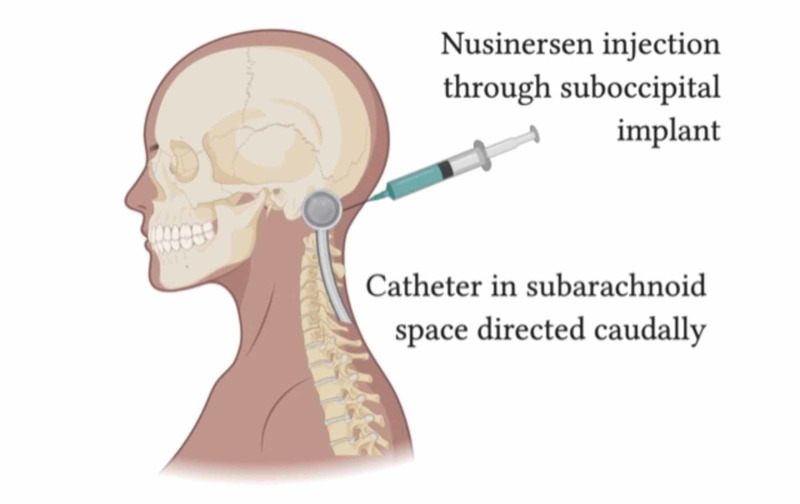
Illustration of injection of Spinraza® through the port with a caudally directed catheter

Secondarily, there might be a physiologic advantage to inject certain treatment modalities into the cervical subarachnoid space, as compared to the lumbar cistern. One could surmise that because SMA affects motor neurons along the length of the spinal cord, a caudally directed cervical catheter, in an upright patient allows for more effective gravity-directed drug delivery down the entire spinal cord (Figure 4). This, in principle, could be a preferable alternative when one is injecting substances such as intrathecal narcotics, intrathecal baclofen, and other intrathecal medications.

In our patient population, there was an overall benefit of this implantable port for injection of Spinraza® with low complication rates. We have done a total of 122 injections without any complications or failures in the port system. The primary complication was CSF leak, which was noted in three patients. One patient developed a transient subcutaneous CSF leak that was resolved without surgical repair after one month. Two of the early patients developed postoperative CSF leak around the port. In both patients, the laminotomy site was explored, and the leaks repaired with muscle and fascial grafts, surgical glue, and fascial cuff closure. These earlier patients had a larger durotomy that was twice the size of the subarachnoid catheter; therefore, CSF could easily leak through the durotomy. Subsequent patients did not have this issue as we made the durotomy significantly smaller.

There are two main limitations to this study. One limitation is that the study is small, with only 15 patients. Although our results provide valuable information regarding the safety and efficacy of Spinraza® injections through a cervical port, the clinical outcomes data from the surgical technique described may not be statistically significant given the small sample size. Additionally, patient identification and data collection were done retrospectively, which comes with inherent biases and flaws, particularly with clinical outcome data. However, the main focus of this article was not to make a clinical comparison, but rather, to show a novel way of Spinraza® injections through an implantable device, which makes injections safer and more efficacious. Clinical outcomes were a secondary objective.

## Conclusions

In patients with complex spinal anatomy, traditional intrathecal drug delivery becomes difficult because of barriers to accessing the subarachnoid space. The procedure and implant process outlined detailed in this article simplifies the CSF access challenge by utilizing an off-label suboccipital port and an off-label lumbar catheter placed in the cervical subarachnoid space. The access of the port/catheter system is minimally invasive and time-efficient and does not require anesthesia and patient repositioning. There are several companies working towards FDA-approved devices that will be similar in function and ease of use. Prior to these devices being commercially available, this system is a good alternative for patients with complex spinal anatomy associated with their spinal muscular atrophy.
